# Polymorphism screening and haplotype analysis of the tryptophan hydroxylase gene *(TPH1) *and association with bipolar affective disorder in Taiwan

**DOI:** 10.1186/1471-2350-6-14

**Published:** 2005-03-31

**Authors:** Te-Jen Lai, Chia-Yen Wu, Hsu-Wen Tsai, Yi-Mei J Lin, H Sunny Sun

**Affiliations:** 1 Institute of Medicine, Chung Shan Medical University, Taichung, Taiwan, Republic of China; 2 Department of Psychiatry, Chung Shan Medical University Hospital, Taichung, Taiwan, Republic of China; 3 Institute of Molecular Medicine, National Cheng Kung University Medical College, Tainan, Taiwan, Republic of China; 4 Institute of Behavioral Medicine, National Cheng Kung University Medical College, Tainan, Taiwan, Republic of China; 5 Institute of Basic Medical Sciences, National Cheng Kung University Medical College, Tainan, Taiwan, Republic of China

## Abstract

**Background:**

Disturbances in serotonin neurotransmission are implicated in the etiology of many psychiatric disorders, including bipolar affective disorder (BPD). The tryptophan hydroxylase gene (*TPH*), which codes for the enzyme catalyzing the rate-limiting step in serotonin biosynthetic pathway, is one of the leading candidate genes for psychiatric and behavioral disorders. In a preliminary study, we found that *TPH1 *intron7 A218C polymorphism was associated with BPD. This study was designed to investigate sequence variants of the *TPH1 *gene in Taiwanese and to test whether the *TPH1 *gene is a susceptibility factor for the BPD.

**Methods:**

Using a systematic approach, we have searched the exons and promoter region of the *TPH1 *gene for sequence variants in Taiwanese Han and have identified five variants, A-1067G, G-347T, T3804A, C27224T, and A27237G. These five variants plus another five taken from the literature and a public database were examined for an association in 108 BPD patients and 103 controls; no association was detected for any of the 10 variants.

**Results:**

Haplotype constructions using these 10 SNPs showed that the 3 most common haplotypes in both patients and controls were identical. One of the fourth common haplotype in the patient group (i.e. GGGAGACCCA) was unique and showed a trend of significance with the disease (P = 0.028). However, the significance was abolished after Bonferroni correction thus suggesting the association is weak. In addition, three haplotype-tagged SNPs (htSNPs) were selected to represent all haplotypes with frequencies larger than 2% in the Taiwanese Han population. The defined *TPH1 *htSNPs significantly reduce the marker number for haplotype analysis thus provides useful information for future association studies in our population.

**Conclusion:**

Results of this study did not support the role of *TPH1 *gene in BPD etiology. As the current studies found the *TPH1 *gene under investigation belongs to the peripheral serotonin system and may link to a cardiac dysfunction phenotype, a second TPH gene that functions predominantly in the brain (i.e., nTPH or TPH2) should be the target for the future association study.

## Background

Bipolar affective disorder (BPD) is a chronic, severe mood disorder characterized by recurrent episodes of mania and depression. It often interferes with the patient's ability to cope with his daily routine and has a high mortality rate from suicide. BPD is estimated to have a lifetime prevalence of 0.1% to 1% in Taiwanese Han [1]. The high prevalence, together with the high frequency of hospitalization, psychosocial impairment, suicide, and substance abuse has made the disease a major public health concern. Although previous studies in epidemiology strongly suggested genetic factors in the etiology of affective disorder [2], establishing the mode of transmission and searching for predisposing genes using linkage analyses have not been successful. Association studies have been useful in mapping genes for complex diseases and are now being applied to many psychiatric traits [3]. However, the success of this approach requires the markers of the candidate gene being used are either the causative variant(s) or in linkage disequilibrium with the causative variant(s) [4]. Results from these studies suggest the involvement of several genes, each of which has a minor effect on the pathogenesis of BPD [5].

Serotonin (also known as 5-hydroxytryptamine) is a major neurotransmitter in the central nervous system (CNS) and is involved in various physiological events, such as mood control, sleep, thermoregulation, learning, and memory [6]. Its role in psychiatric disorders is well documented [7]. Disruption of serotonergic function has been implicated in the pathogenesis of many psychiatric disorders, including BPD [8,9], making genes involved in serotonin transmission and its metabolic pathway good candidates for involvement in BPD pathogenesis.

Tryptophan hydroxylase (TPH) catalyzes the biopterin-dependent monooxygenation of tryptophan to 5-hydroxytryptophan, which is subsequently decarboxylated to form the neurotransmitter, serotonin [10]. Two tryptophan hydroxylase isoforms have been identified [11-13] and the expression of TPH1 and TPH2 were found to be mainly in the pineal gland [14] as well as in the peripheral tissues (duodenum, kidney or lung; [15]) and in the brain stem [12,15], respectively. The *TPH1 *gene has been the subject of intensive study regarding its possible involvement in many psychiatric and behavioral traits [16]; And an increasing number of investigations have studied the *TPH2 *gene effect in association with several psychiatric and behavioral traits including antidepressant response [17] and major depression [18].

The *TPH1 *gene, which has been mapped to human chromosome 11p15.3-p14 [13], spans a region of 29 kilobases and consists of 11 exons [19]. Numerous mRNA species are transcribed from a single promoter by alternative splicing of three exons (1A, 1B, and 1C) in the 5'- untranslated region (UTR) [19,20] and two exons (11A and 11B) in the 3'UTR [19,20]. The structure of the gene and the sequence of the coding region are very similar to those for the genes for human tyrosine hydroxylase and phenylalanine hydroxylase, which belong to the super-family of aromatic amine acid hydroxylases. Polymorphisms within intron 7 of the *TPH1 *gene have been implicated to be associated with several psychiatric conditions such as BPD [21], suicidal behavior and alcoholism [22], and aggression-related traits [23]. Furthermore, the *TPH1 *intron 7 polymorphism was associated with the 5-HIAA level in both impulsive Finnish alcoholics [24] and healthy men [25] thus suggesting that *TPH1 *genotypes may participate in the regulation of serotonin turnover rate in the central nervous system. However, such associations were not found in other studies involving suicidal behavior [26], BPD and mood disorders [27,28], and attention-deficit hyperactivity disorder [29]. These studies indicate that the role of TPH1 in predisposing different psychiatric traits varies and could be modified by other factors like ethnicity or gene-to-gene interactions [30]. Preliminary study in our laboratory has found an allelic association (P = 0.03) between the *TPH1 *intron 7 A218C polymorphism and BPD in 42 Taiwanese bipolar patients and 70 matched healthy controls [31]. Results from these studies suggest that *TPH1 *may play a role in regulating serotonin metabolism. More recently, sertraline, a selective serotonin reuptake inhibitor and an effective antidepressant, has been shown to up-regulate *TPH1 *expression and serotonin synthesis [32], suggesting one of the mechanism for its pharmacological action in antidepressive therapy.

To provide information of the *TPH1 *gene for future genetic analysis of disorders involved the disturbances in the serotonergic system in Taiwan, we have systematically screened all 11 exons and the promoter region of the human *TPH1 *gene for common sequence polymorphisms and have identified 5 single nucleotide polymorphisms (SNPs). In addition, we carried out a case-control study of 108 BPD type I patients and 103 normal controls looking for an association between BPD and these 5 SNPs plus 5 others taken from the literatures [33,34] and a public SNP database [35], tested both as individual SNPs and as the haplotypes.

## Methods

### Subjects

Since 1998, probands were recruited from bipolar outpatients in the Chung Shan Medical University Hospital and the Taichung Rehabilitation Hospital in Taiwan. Controls were subjects from local volunteer blood donors who have no family or personal history of major affective disorder and other psychiatric disorders were matched to cases on the basis of ethnic or geographic origin, sex, and age. The gender ratios are identical in both case and control groups (male to female is 53% to 47 %) and the age of populations are 38 ± 13.7 and 26.4 ± 7.9 in case and control groups, respectively. The age of onset is 28.4 ± 12.7 in all patients and is 30.3 ± 14.5 and 26.4 ± 10.2 in male and female patients, respectively. Clinical interviews were conducted by experienced psychiatrists (leading by Dr. Lai) for all subjects after the study procedure had been fully explained and information on general demographic data, such as age, sex, and ethnicity, was obtained. Patients assessment was purely by direct clinical interview by the treating clinician according the procedure described in the DSM-IV diagnoses of lifetime Major depressive disorder and bipolar I. Additional information required to reach diagnosis was also collected from all clinical and hospital records where available but the comorbidity with other psychiatric/neurological disorders or medical problems were not considered as an inclusion/exclusion criteria. This study was approved by the University Ethics Committee and written informed consent was obtained from all participants. In total, 108 BPD and 103 controls (all Taiwanese Han) were recruited for this study.

### DNA preparation

Approximately 10 ml of peripheral blood was collected from the recruits using EDTA anticoagulant venous blood tubes, and DNA prepared using a BIO-RAD InstaGene™ Whole Blood Kit (Bio-Rad Laboratory, Hercules, CA) according to the manufacturer's protocol.

### Primer design for searching for, and typing of, TPH1 gene polymorphisms

All primers used were designed using the Primer3 program [36] or OLIGO 5 Primer Analysis Software (Molecular Biology Insights, Inc., cascade. CO., USA). The primers used to screen the *TPH1 *gene (Table 1) were designed using the published sequence (GenBank accession number: AC005728). The primers used in exon-wide SNP scanning were designed from the intronic sequence roughly 50 bp upstream and downstream of each exon to amplify the entire exon sequences. In addition, overlapping fragments covering from 1,151 bp upstream of the 5' promoter region to 1,572 bp downstream of the 3'UTR were also amplified to screen for polymorphisms within these regions. The forward and reverse primers for base excision sequence scanning (BESS) product amplification were labeled, respectively, with fluorescent 6-FAM and HEX to facilitate variant detection.

### SNP identification in the coding and regulatory regions of the TPH1 gene

In order to identify all SNPs in the coding and regulatory regions of the *TPH1 *gene, we designed primers to generate PCR products for use in SNP identification using the BESS Base Reader Kit (Epicentre Technologies, Madison, WI) according to the manufacturer's protocol. Each identified SNP was sequenced to confirm the sequence variant. The screening panel included 50 unrelated subjects with or without BPD.

The BESS T & G Base Reader Kit, which identifies all types of point mutation, deletion, insertion, repeat expansion, and frameshift mutation at sites involving thymine or guanine, was used to systematically search for sequence variants in pooled samples. DNA sequence variants are detected by cleavage of the amplification products at modified nucleotides, generating a defined series of fragments which can be easily separated on a standard sequencing gel and detected using a fluorescent dye detection system. Briefly, PCR products were generated using FAM-labeled forward primers and HEX-labeled reverse primers. PCR amplification was performed in a 25 μl volume containing 1 unit of Taq polymerase, 1× PCR buffer, 0.2 μM of each labeled primer, an appropriate concentration of MgCl_2_, 200 μM BESS T/G Scan dNTP Mix, and 50 ng of genomic DNA. Thermal cycling conditions were a pre-denaturation of 3 min at 94°C; 35 cycles of 30 sec at 94°C, 30 sec at the appropriate annealing temperature indicated in Table 1, and 30 sec of extension at 72°C; and a final extension at 72°C for 5 min.

For the excision reaction, 5 μl of the amplification reaction was mixed with 1 μl of the BESS T/G-Scan Excision Enzyme Mix and 1 μl of 10X BESS T/G-Scan Excision Enzyme Buffer, the mixture incubated for 30 minutes at 37°C, and the reaction stopped by adding 5 μl of Stop/Loading Buffer. One microliter of the excision reaction products was mixed with gel loading solution containing 12 μl of formamide and 8 μl of GENESCAN™-500 size standards (Applied Biosystems, Forster City, CA, USA), then the mixture was denatured for 5 min at 95°C, loaded onto a capillary polymer of the ABI 310 Genetic Analyzer, and run for 30 min for size separation. Analysis was performed using the GENESCAN 672 program (Applied Biosystems).

To reduce the cost and speed up the process, a pooling methodology was used. A preliminary test indicated that the sensitivity of BESS T/G-Scan analysis allowed the detection of alleles with a frequency in the population greater than 4.5 % (data not shown), i.e., The assay can recognize 1 heterozygous individual in a DNA pool from this individual and 9 homozygotes (equivalent to a minor allele with a frequency of 5%). DNA from 10 individuals was therefore pooled (10 ng/μl of DNA from each individual) and five such pools, representing 100 chromosomes, were prepared from 50 randomly selected subjects for polymorphism identification.

### Selection of SNP markers for the TPH1 gene

In addition to the SNPs identified in the present study, five SNP markers within and flanking the human *TPH1 *gene were also selected to test the association with BPD. Based on the literature [34] and a public SNP database [35], SNP markers roughly 10 kb apart and with a relatively high minor allele frequency were selected. The *TPH1 *intron 7 A218C polymorphism, which has been suggested to be associated with Taiwanese BPD in our previously work, also has been included in the present study (i.e. in7SNP1; A20004C). The genomic localizations of the 10 SNPs examined relative to the transcription start site are given in Table 1.

### SNP genotyping

Except for the intron 7 A218C polymorphism, which was genotyped using a modified Amplification Refractory Mutation System (ARMS), all other SNPs of the *TPH1 *gene were genotyped using a multiple SNP genotyping system which involves multiplex PCR and multiple single base extensions (MSBE). Briefly, two multiplex PCR reactions were performed. One was in a 20 μl volume containing 70 ng of genomic DNA, 2.4 μl of primer mix (0.1 μM of the primer pairs for 5'flankingSNP3, in1SNP1, in2SNP1, and in6SNP1, and 0.2 μM of the primer-pair for in3SNP1), 400 μM dNTPs, 1× PCR buffer, 1.5 mM MgCl_2_, and 1 U of Taq polymerase. The conditions used were an initial denaturation step of 5 min at 95°C, 30 cycles of amplification (30 s at 95°C, 60 s at 51°C, 90 s at 72°C), and a final extension step of 10 min at 72°C. The other multiplex PCR was performed under the same conditions, but using 0.8 μl of primer mix containing 0.1 μM of the primer pairs for 5'flankingSNP1, 5'flankingSNP2, 3'UTRSNP1, and 3'UTRSNP2. A 4 μl aliquot of the PCR products was treated with 5 U of shrimp alkaline phosphatase (SAP) and 0.1 U of exonuclease I in a total volume of 10 μl to remove primers and unincorporated dNTPs; the reaction was carried out at 37°C for 1 hour, then terminated by incubation at 72°C for 15 minutes to inactivate the enzymes. The multiplex SBE reaction was carried out using SNP-specific primers and fluorescent-labeled terminators (the ABI PRISM SNaPshot Multiplex Kit). The short-extension reaction was performed on a thermal cycling machine for 25 cycles of 10 s at 96°C, 5 s at 50°C, and 30 s at 60°C. After short-extension, excess ddNTPs were removed from the SBE products by addition of 0.5 U of SAP to the reaction mixture and incubation at 37°C for 1 hour. The purified SBE products were electrophoresed on a ABI PRISM 310 Genetic Analyzer and analyzed using GeneScan software (ABI PRISM).

### Statistical analysis

The chi-squared test for allelic and genotypic distributions between patients and controls was performed using the CROSSTAB program implemented by SPSS. The Hardy-Weinberg equilibrium was analyzed using the HWE program, version 2.33 [37]. Pairwise LD coefficients D' [38] among the 10 SNPs were estimated and statistical significances were determined by using the SNP Alyze^® ^program (SNP and Disease Association Analysis software; Dynacom Co., Ltd. Kanagawa, Japan). In addition, the PHASE 2.0 program [39,40] was used to construct haplotypes and perform a case-control permutation test, then the Fisher's exact test was applied to test differences in haplotype frequencies between cases and controls. All Fisher's exact tests (two tails) and estimation of the odds ratio of BPD associated with a particular haplotype were performed using the PROC FREQ program implemented by SAS package (SAS Institute Inc., Cary, NC, USA). Haplotype-tag SNPs were selected using SNPtagger software [41]. To consider the multiple comparisons, a Bonferroni correction was applied in this study thus the p value for reaching significance is 0.005 for 10 SNPs. In addition, the potential confounders such as personality disorders, substance abuses or organic disorders were not considered in the statistic analyses.

## Results

### Detection of TPH1 gene polymorphisms in the Taiwanese Han population

To detect SNPs in the human *TPH1 *gene, we used the enzymatic cleavage approach to screen all 11 exons, parts of neighboring introns, and the promoter of the gene in 30 unrelated healthy and 20 BPD probands from a previously recruited population [31]. No sequence variation in the coding region of the *TPH1 *gene was identified. However, screening for nucleotide variants up to -1,151 bases in the promoter region and down to 1,572 bases in the 3'UTR identified five SNPs. Two, A-1067G (5'flankingSNP2) and G-347T (5'flankingSNP3), were found in the promoter region. Of the remaining 3 SNPs, T3804A (in1SNP1) was located in intron 1/exon 1c, while C27224T (3'UTRSNP1) and A27237G (3'UTRSNP2) were located in the 3'UTR of exon 11.

### SNP genotyping and single locus association analysis

For further association analysis, all 5 SNPs identified in this study, plus one polymorphism in the promoter region (5'flankingSNP1;[33]) and four selected from the dbSNP database (in2SNP1, in3SNP1, in6SNP1, and in7SNP1), were genotyped with MSBE or ARMS (Table 1). All genotypes were in Hardy-Weinberg equilibrium (HWE), except for the G-347 T marker in the promoter region that had departure from the HWE in both controls (p = 0.024) and patients (p = 0.024). Genotype coverage was generally good and about 90% of the subjects were successfully typed.

All 10 SNPs were genotyped and have been analyzed by Fisher's exact test between the case and control groups. No association can be obtained between any of the *TPH1 *gene polymorphisms and BPD. The genotypic and allelic distributions of each marker are listed in Table 2.

### Pairwise linkage disequilibrium (LD) measurement and haplotype analyses

The size of the human *TPH1 *gene is about 29 kb and the LD between all pairs of 10 SNP markers in all subjects was estimated using the SNP Alyze^® ^program (Table 3). The data above and below the diagonal line in Table 3 show, respectively, the results of the pairwise LD analysis, as indicated by the D' [38]; a higher value indicating a higher degree of LD), and the p value for the chi-squared test. As shown in Table 3, the D' values ranges from 0.032 to 1 and the majority of the D' value is greater than 0.5. These data suggested that sequence variants within the *TPH1 *gene are in strong disequilibrium (the upper diagonal of the Table 3). Furthermore, as indicated by the lower diagonal of Table 3, statistically significant LD was detected for almost all marker pairs among these SNPs. Few exceptions were observed for marker pairs T3840A / A-1067G, A27237 / G-347T, and A27237 / GA12517C in which the D' values are 0.076, 0.032, and 0.039, respectively.

Haplotypes constructed using the PHASE 2.0 program [39] revealed a total of 70 haplotypes in combined controls and patients (data not shown). Among these haplotypes, the majority is in very low frequency but the four major haplotypes with frequency greater than 2% are found in 59.8% of the total population. Estimation of haplotype distribution and the Fisher's exact test values for testing the significance of differences in individual haplotype frequencies between case and control groups for the haplotypes with frequency > 2% in at least one group were listed in Table 4. Additional test was performed to assess overall haplotype frequency profile differences, rather than frequency differences for a specific haplotype, and p value estimated from 1000 permutation tests was also obtained (Table 4). Although a trend of significance was detected for the GGGAGACCCA haplotype between the case and control groups (p = 0.028) and few haplotypes showed slightly increased risks in which the value of odds ratios are larger than 2, the significances were abolished after Bonferroni correction for multiple tests thus suggested the effect is minor (Table 4). Furthermore, the overall comparison for all haplotypes between two groups was not significant (p = 0.082) and the p value from 1000 time permutation test was also not significant (p = 0.602).

### Haplotype tag selection

Selecting haplotype-tag SNPs (ht-SNPs) can systematically and efficiently refine the process of haplotype construction and reduce the marker number for genotyping. In this study, three htSNPs (5'flankingSNP1, in2SNP1, in6SNP1) were selected to represent all haplotypes with frequencies larger than 2% using SNPtagger software [41] (Table 5). Results from the htSNP analysis suggest that the information obtained by examining three SNPs (instead of 10) should be sufficient for genetic analysis, since these can be used to construct and represent all common haplotypes with frequency >2%. Five haplotypes were constructed using these 3 htSNPs and statistical analyses detected no differences in haplotype distributions between cases and controls (Table 5).

## Discussion

The involvement of the *TPH1 *gene in the pathogenesis of affective disorder is supported by several lines of evidence. Previous studies have reported a significant association between *TPH1 *intron 7 A218C polymorphism and BPD in the French population [21]. In addition, two current meta-analyses confirmed the significant association of suicide-related behavior with *TPH1 *A218 polymorphism [42,43] and suggested that the A allele has a dose-dependent effect on the risk of suicidal behavior [43]. Since these results suggested that *TPH1 *could be a strong candidate for involvement in BPD, we have systematically searched all the exons and promoter region of the gene for SNPs. Five polymorphisms were identified, one of which, 3'UTRSNP2, is a novel polymorphism not previously reported. The only sequence variant found in all *TPH1 *exons was in the exon 1c/intron1 region (T3804A, dbSNP ID: rs623580), but the polymorphism is within the 5'-UTR and therefore does not result in an amino acid substitution. This finding is consistent with a previous report in which no coding sequence variant of *TPH1 *was detected in Americans (Indians or Caucasians), Italians, and Finns [44]. These results demonstrate the highly conserved nature of the human *TPH1 *gene. The exon 1c/intron 1 T3804A polymorphism has been previously reported and has an estimated minor allele frequency of 0.001 in the Swiss population [34,45].

The five identified SNPs, together with five additional SNPs taken from the literature and a public database, were tested for an association with BPD in Taiwanese patients. In general, the alleles in the *TPH1 *gene were common, with minor allele frequencies ranging from 21% to 49% and from 22% to 49% in cases and controls, respectively. The genotypic frequencies of all markers, except the G-347T (5'flankingSNP3) marker in the promoter region, showed Hardy-Weinberg equilibrium (HWE) in both populations. The departure from HWE of 5'flankingSNP3 was observed in both controls (p = 0.024) and patients (p = 0.024). A separate study in our lab indicated that the location of the G-347T polymorphism is on the transcriptional repressor GATA1 binding site [46] and alteration of alleles indeed change promoter activity in a luciferase reporter gene system [47]. The departure from HWE could be explained by the negative selection of the homozygous TT individuals. And the presence of very low frequency of TT genotype in the population may be the reason for the impossibility of detecting any association. Despite the strong functional role of the *TPH1 *G-347T polymorphism, no association was detected between any of the polymorphisms and BPD in Taiwanese.

The extent and distribution of LD in humans has been a hot topic, especially for gene mapping of complex diseases. In this study, significant LD, as measured by the D' and P values using the SNP Alyze^® ^program, could be detected between T-1721G and C27224T polymorphisms of the *TPH1 *gene, which are separated by roughly 29 kb (Table 3). Because LD-induced association between multiple loci that harbor disease-predisposing alleles can be identified by haplotype-based analyses [48], haplotypes were constructed and their frequencies were compared between cases and controls. Haplotype distributions among 10 *TPH1 *SNPs were estimated and twelve haplotypes with frequency larger than 2 % in at least one group were listed (Table 4). The three most common haplotypes were identical in both groups and were found in 58 % and 56.7 % of the patients and controls, respectively. Although significant difference in haplotype distribution was seen in one comparison, the association was weak and was lost after the Bonferroni correction for multiple tests. Additional permutation tests for haplotype distributions in case and control groups were performed and result indicated no differences in haplotype profile between two groups (p value = 0.602). The data suggest that both BPD patients and controls are actually from the same population thus it implies the *TPH1 *gene may not be related to BPD etiologies.

Positive association between *TPH1 *intron 7 polymorphism and BPD was identified in our previous study [31], but the replication with extended samples has failed to confirm this association in both single-locus and haplotype analyses. One possible reason for this discrepancy could be at the sampling bias as the age of controls in the extended population is much younger than the cases. Since mood disorders may occur late in life, the sampling bias represents a major limitation of this study. Another possible explanation may be that the *TPH1 *gene has only a minor effect on BPD etiology, this effect being missed when heterogeneous samples are used. Alternatively, the positive association we obtained initially could be a false positive outcome from a very small sample size being used. Recently, studies of Tph1 (the original Tph) knockout mice were found to express normal amounts of serotonin in brain, but not in the periphery [11], and resulted in a cardiac dysfunction phenotype [49]. Follow up studies found that the second tryptophan hydroxylase (TPH2, also known as nTPH) gene is predominantly expressed in the brain stem, while the classical TPH1 is expressed in the pineal gland [14] and peripheral tissues (duodenum, kidney or lung;[15]. The amount of TPH2 mRNA expression in individual raphe cells was estimated to be approximately 2.5-fold greater than the level of TPH1 expression in pinealocytes [14]. These findings have changed the consideration of linking polymorphism of *TPH1 *gene with various psychiatric diseases [12] and perhaps, could explain the lack of association between the TPH1 gene and BPD obtained in this study. Whether these two paralog proteins are regulated independently or if they have distinct functions in the brain are still under investigation; studies to establish connections between *TPH2 *gene and various psychiatric diseases including BPD are on going and should provide more insights regarding the TPH2 function in the brain.

Studies on sequence variations in the human genome have revealed that the human genome can be parsed objectively into haplotype blocks, with limited diversity within each block [50,51]. Johnson et al. [52] determined the extended haplotype at any given locus in a population to identify the SNPs in a gene or a LD region, information that is essential for association studies. The so-called "haplotype tag SNPs (htSNPs)" capture the majority of haplotype diversity within a region and thus represent the minimal number of markers that need to be typed to define the common haplotypes (higher than 5% frequency in the population). In this study, we used a publicly program to define htSNPs that represent all haplotypes of the *TPH1 *locus with a frequency > 2% [41]. Three htSNPs, corresponding to all common haplotypes were generated using SNPtagger software [53] (Table 5). Although further analysis using haplotypes constructed with htSNPs revealed no differences in frequency distributions between cases and controls (Table 5), the htSNPs selected for human *TPH1 *gene significantly reduce the number of markers required for genotype analysis (in this case from 10 to 3 SNPs), which may be useful in other studies on the Taiwanese Han population.

## Conclusion

Bipolar disorder is a complex genetic disorder with a spectrum of phenotype is associated with bipolar susceptibility genes. Since the subject assessment in this study was only by direct clinical interview according to the DSM-IV but not supported by any other standard tools like the SCID, it is worth noting the potential weakness of the study. The simple assessment procedure without dimensional measures of other psychopathological features also limits the possibility to detect association with particular attribute related to the disease. In summary, results from both single-locus and haplotype analyses did not support the role of *TPH1 *gene in BPD etiology. Furthermore, our data indicated significant LD within this 29 Kb interval of the *TPH1 *gene in Taiwanese population. Since haplotype frequencies and LD often differ between racial/ethnic groups, the three htSNPs identified in this study should be beneficial in future application for genetic study in Taiwanese population.

## Competing interests

The author(s) declare that they have no competing interests.

## Authors' contributions

TJL was in charge of clinical examination and provided clinical samples. CYW designed polymorphism screening methods and identified all *TPH1 *polymorphisms in Taiwanese. HWT conducted genotyping experiments and provided statistical analysis. YJL contributed to genotyping data analysis. HSS provided overall study design, analysis, and drafted the manuscript. All authors read and approved the final manuscript.

## Pre-publication history

The pre-publication history for this paper can be accessed here:



## Figures and Tables

**Table 1:** Summary of the 10 human *TPH1 *SNPs genotyped in this study^a^

SNP name (Genomic localization)^b^	Position in the gene	SNP ID^c^	PCR primers and short-extension probe^e^
5'flankingSNP1 (T-1721G)	5' flanking region	SNP000574351	F: 5'-ctgttcttttggtgtcctc-3' R:5'-gctcctggcacttaacata-3' P: 5'-taatttctttcatgagtattttatagtt
5'flankingSNP2 (A-1067G)	5' flanking region	SNP000574353	F: 5'-ctgttcttttggtgtcctc-3' R: 5'-gctcctggcacttaacata-3' P: 5'-ttttttgctgagtatggatgtactttaaagctcagga
5'flankingSNP3 (G-347T)	5' flanking region	SNP000574354	F: 5'-cgataataggcgttatcttg-3' R: 5'-ctcaatctctgcgtgtatct-3' P: 5'-tcaggactgggctattaaatagcccagaagcacagaga
in1SNP1 (T3804A)	Intron 1 (exon 1c)	rs623580	F: 5'-taattatcctccctccaagt-3' R: 5'-cttacccattcaattaccac-3' P: 5'-agagtatgggcgacgttgtccta
in2SNP1 (G7465A)	Intron 2	rs684302	F: 5'-tgctcttatatgtcttttcaagt-3' R: 5'-gagagatggagcaaaacac-3' P: 5'-ttaaataaaatacctgtatgtcttcttccatca
in3SNP1 (A12517C)	Intron 3	rs211105	F: 5'-tcaggaaaacagaagggta-3' R: 5'-ggtaaattgccctatttctaa-3' P: 5'-aggtggcaaagacaaatgatttctaagatcttttccatcggc
in6SNP1 (C18626G)	Intron 6	rs2237907	F: 5'-gggaagaaattatgtaagtgg-3' R: 5'-gaaatgttccatatctgtgc-3' P: 5'-ttgtaatgcacacaaaactgaaagctgatctcttagggtctggagc
in7SNP1 (A20004C)^d^	Intron 7	rs1800532	CF: 5'-acccacctacactttcctc-3' CR: 5'-taattgacaacctattaggttc-3' AR: 5'-agcacatgtgaagcatttag-3' AF: 5'-cctatgctcagaatagcagctct-3'
3'UTRSNP1 (C27224T)	3' UTR	rs2108977	F: 5'-cacttgaatatcacagtccatc-3' R: 5'-gcttacagtagatttccttgc-3' P: 5'-tacatttgatggtaaatagatgctagctaatct
3'UTRSNP2 (A27237G)	3' UTR	New	F: 5'-cacttgaatatcacagtccatc-3' R: 5'-gcttacagtagatttccttgc-3' P: 5'-aactataaatcagataatcaata

a. in7SNP1 was genotyped using ARMS, while the other SNPs were genotyped using MSBE.

b. Genomic localizations of SNPs are given in bp relative to the transcription start site (position 0).

c. The ID for 5'flankingSNP1, 5'flankingSNP2, and 5'flankingSNP3 are from HGVbase . The ID for in1SNP1, in2SNP1, in3SNP1, in6SNP1, in7SNP1, 3'UTRSNP1, and 3'UTRSNP2 are from dbSNP .

d. The A20004C is known as intron7 A218C polymorphism.

e. All PCR reactions were performed at the same annealing temperature (53°C). F: forward primer, R: reverse primer, P: short extension probe.

**Table 2:** Genotype and allele frequencies of the *TPH1 *gene polymorphisms.

Marker	Group^a^	Genotype^b ^(frequency)					Allele (frequency)			
			
	(Total Number)	11	12	22	*X*^2^	*p *value	1	2	*X*^2^	p value
T-1721G	N(94)	54(.57)	37(.39)	3(.03)	2.79	0.248	145(.77)	43(.23)	0.36	0.548
	P(92)	53(.58)	31(.34)	8(.09)			137(.75)	47(.26)		
A-1067G	N(90)	53(.59)	34(.38)	3(.03)	1.62	0.446	140(.78)	40(.22)	0.74	0.389
	P(92)	51(.55)	34(.37)	7(.08)			136(.74)	48(.26)		
G-347T	N(102)	58(.57)	43(.42)	1(.01)	0.048	0.976	159(.78)	45(.22)	0.037	0.847
	P(94)	52(.55)	41(.44)	1(.01)			145(.77)	43(.23)		
T3804A	N(101)	56(.55)	38(.38)	7(.07)	2.17	0.304	152(.74)	52(.26)	0.015	0.946
	P(96)	49(.51)	44(.46)	3(.03)			142(.74)	50(.26)		
G7465A	N(100)	31(.31)	43(.43)	26(.26)	0.46	0.793	105(.53)	95(.48)	0.439	0.508
	P(74)	25(.34)	33(.45)	16(.22)			83(.56)	65(.44)		
A12517C	N(101)	58(.57)	39(.39)	4(.04)	0.232	0.890	155(.77)	47(.23)	0.207	0.649
	P(96)	58(.60)	35(.37)	3(.03)			151(.79)	41(.21)		
C18626G	N(100)	28(.28)	47(.47)	25(.25)	1.24	0.538	103(.52)	97(.49)	0.392	0.531
	P(71)	25(.35)	28(.39)	18(.25)			78(.55)	64(.45)		
A20004C	N(102)	40(.39)	45(.44)	17(.17)	1.726	0.422	125(.61)	79(.39)	1.795	0.18
	P(89)	29(.32)	39(.44)	21(.24)			97(.54)	81(.46)		
C27224T	N(95)	31(.33)	49(.52)	15(.16)	3.58	0.167	111(.58)	79(.42)	2.13	0.144
	P(80)	23(.29)	35(.44)	22(.28)			81(.51)	79(.49)		
A27237G	N(95)	49(.52)	40(.42)	6(.06)	0.57	0.751	138(.73)	52(.27)	0.677	0.411
	P(78)	37(.47)	34(.44)	7(.09)			107(.69)	49(.34)		

a. N indicates the matched normal controls, P indicates the bipolar patients.

b. 1 represents the major allele and 2 the minor allele.

**Table 3:** Pairwise linkage disequilibrium indicated by the D' value (above the diagonal)^*a *^and statistical significance indicated by the p value (below the diagonal)^*b *^for the ten *TPH1 *SNPs.

	T-1721G	A-1067G	G-347T	T3840A	G7465A	A12517C	C18626G	A20004C	C27224T	A27237G
T-1721G		1.000	0.911	0.129	0.834	0.505	0.601	0.726	0.329	0.177
A-1067G	<0.001		0.913	0.076	0.863	0.537	0.655	0.722	0.391	0.274
G-347T	<0.001	<0.001		0.121	0.840	0.450	0.645	0.655	0.447	0.032
T3840A	0.020	**0.166**	0.033		0.969	0.575	0.870	0.652	0.512	0.574
G7465A	<0.001	<0.001	<0.001	<0.001		0.685	0.711	0.826	0.741	0.929
A12517C	<0.001	<0.001	<0.001	<0.001	<0.001		0.684	0.320	0.663	0.039
C18626G	<0.001	<0.001	<0.001	<0.001	<0.001	<0.001		0.765	0.684	0.780
A20004C	<0.001	<0.001	<0.001	<0.001	<0.001	<0.001	<0.001		0.624	0.625
C27224T	<0.001	<0.001	<0.001	<0.001	<0.001	<0.001	<0.001	<0.001		0.956
A27237G	**0.203**	**0.096**	**0.840**	<0.001	<0.001	**0.809**	<0.001	<0.001	<0.001	

^a ^Standardized linkage disequlibrium coefficient (absolute D' value) of Lewontin was shown.

^b ^Significant levels based on the chi-squared distribution to test whether the disequilibrium value differs from zero (one degree of freedom). Bold and underline indicate no significant LD at the *p *< 0.05 level.

**Table 4:** Haplotype frequency estimation and test statistics between case and control.

**Haplotype^a^**	**Patient**	**Normal**	**Fisher's Exact Test (*P *value)^b^**	**OR^b,c,d^**	**95% CI^d^**
**TAGTAAGACA**	0.340	0.359	0.755	0.919	0.61–1.38
**TAGAGACCTG**	0.145	0.140	1.000	1.035	0.59–1.80
**GGTTGCCCTA**	0.095	0.068	0.366	1.439	0.70–2.96
**TAGTAACACA**	0.010	0.039	0.105	0.250	0.05–1.19
**TAGTAAGATA**	0.025	0.010	0.278	2.615	0.50–13.64
**TAGTGCGACA**	0.025	0.015	0.498	1.735	0.41–7.36
**GGGAGACCCA**	0.025	0	0.028	--	--
**TAGTAACCTG**	0.020	0	0.058	--	--
**TAGTGACCTG**	0.020	0.010	0.443	2.081	0.38–11.49
**TAGAGACATG**	0.020	0.019	1.000	1.031	0.25–4.18
**GGTTGCCCTG**	0.020	0.010	0.443	2.082	0.38–11.49
**GGTAGACCCA**	0.015	0.024	0.724	0.612	0.14–2.59
**Others**	0.240	0.306	0.149	0.717	0.46–1.11
Total			0.082		
***P *value of permutation test**	**0.602**				

a. The order of the markers, shown 5' → 3', is (left to right) T-1721G, A-1067G, G-347T, T3804A, G7465A, A12517C, C18626G, A20004C, C27224T, A27237G.

b. Chi-square, Fisher's Exact Test and Odds Ratio were calculated by using the SAS program.

c. The Odds Ratio could not be calculated for the haplotype GGGAGACCCA and TAGTAACCTG, because of the zero value in the population.

d. OR: odds Ratio; CI: confidence Interval.

**Table 5:** Haplotype frequency and test statistics between cases and controls using htSNPs.

**Haplotypes from htSNPs^a^**	**Represented haplotypes from 10 SNPs^a^**	**Patient (frequency)**	**Normal**	**Fisher's Exact Test (*P *value)^b^**	**OR^a,c^**	**95% CI^c^**
**TAG**	**TAGTAAGACA** **TAGTAAGATA**	0.365	0.369	1.000	0.983	0.66–1.47
**TGC**	**TAGAGACCTG** **TAGTGACCTG** **TAGTGACATG**	0.185	0.169	0.699	1.109	0.66–1.85
**GGC**	**GGTTGCCCTA** **GGGAGACCCA** **GGTTGCCCTG** **GGTAGACCCA**	0.155	0.102	0.137	1.616	0.89–2.92
**TAC**	**TAGTAACACA** **TAGTAACCTG**	0.030	0.039	0.787	0.765	0.26–2.25
**TGG**	**TAGCGCGACA**	0.025	0.015	0.498	1.735	0.41–7.36
**Others**		0.240	0.306	0.149	0.717	0.46–1.11
**Total**				0.446		

a. SNPs in bold and dark color are htSNPs selected from common haplotypes using the SNPtagger program.

b. Chi-square, Fisher's Exact Test and Odds Ratio were calculated by using the SAS program.

c. OR: odds Ratio; CI: confidence Interval.
